# Resistance training in older adults: are the gains worth the strain?

**DOI:** 10.1016/j.ajpc.2026.101569

**Published:** 2026-03-19

**Authors:** Bradley J. Petek, Nathaniel Moulson, Jason V. Tso

**Affiliations:** a Sports Cardiology Program, Knight Cardiovascular Institute, Oregon Health & Science University, Portland, OR, USA; b Division of Cardiology and Sports Cardiology BC, University of British Columbia, Vancouver, BC, Canada; c Division of Cardiovascular Medicine, Stanford Center for Inherited Cardiovascular Disease, Stanford, CA, USA; d Division of Cardiovascular Medicine, Stanford University School of Medicine, Stanford, CA, USA

**Keywords:** Weightlifting, Exercise, Physical activity, Frailty, Blood pressure

Regular exercise training improves longevity and well-being. Current national physical activity guidelines in the United States (US) recommend resistance training ≥2 days weekly. Regular resistance training has profound effects on an individual’s health and well-being including lowering mortality by ∼15%, as well as improving blood pressure, lipid profiles, glycemic control, body composition, and physical functioning [1]. While individuals meeting resistance training guidelines in the US has increased over the past decades, only 28% of US adults in 2018 reported meeting physical activity standards for resistance training, including 19% of adults ≥65 years old (Fig. 1) [2].

**Fig. 1:**
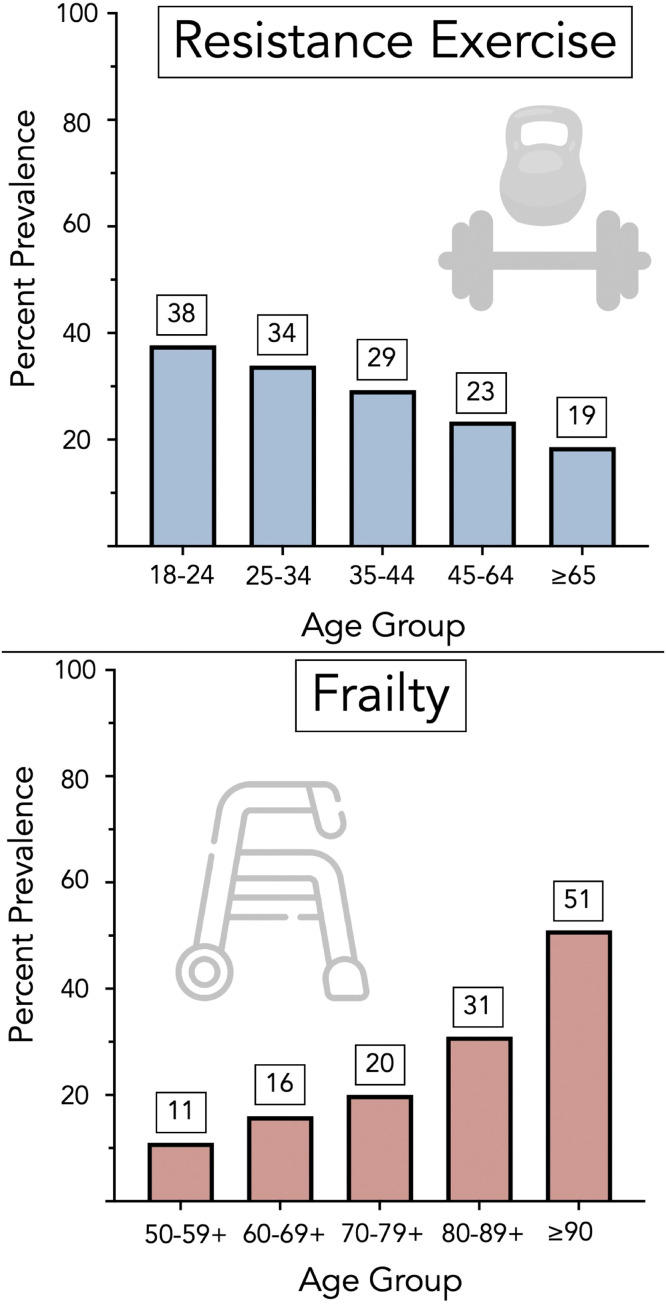
Percent Prevalence of Individuals in the United States Meeting Guideline Recommendations for Resistance Exercise per Week and Individuals Around the World Meeting Definitions for Frailty Stratified by Age Group Top: Figure was creating using the reported prevalence of individuals in the United States meeting minimum recommendations for resistance exercise in the year 2018 [2]. Bottom: Figure was created using data from a systematic review and meta-analysis of patients from 62 countries that reported prevalence estimates for frailty [6].

In this issue of the *American Journal of Preventive Cardiology*, Laddu et al. present the INERTIA study which is a randomized clinical trial of a resistance exercise intervention in older adults (≥60 years old) with low muscular strength without a known history of cardiovascular disease [3]. Investigators enrolled 71 older adults (average age 70±6 years, 76% female) from February 2020 to July 2023, and randomized them in a 2:1 fashion to a 12-week progressive resistance training (PRT) intervention (*n* = 50) or an assessment-only control group (*n* = 21). A total of 61/71 (86%) participants completed follow-up in the study. The PRT group had greater reductions in diastolic blood pressure compared with the control group (DBP, −4.2 mmHg [95%CI −8.3, −0.1]) but there was no significant difference in reduction of systolic blood pressure (SBP, −5.4 mmHg [95%CI −13.0, 2.2]). Within the PRT group, both DBP (−3.9 mmHg [95%CI −6.8, −0.9]) and SBP (−5.6 mmHg [95%CI −11.1, −0.1]) significantly decreased throughout the study period while there were minimal changes in controls. For secondary outcomes, strength gains were greater in the PRT group than controls (chest press, 11.0 kg; leg press, 63.6 kg, *p* < 0.001) and there were no significant differences in between-group changes in the short physical performance battery (SPPB, PRT: 2 points, controls: 1.2 points).

The authors of this study are to be commended for performing this important work. Randomized exercise intervention trials are inherently difficult to design and implement, and this study focuses on a growing population around the world, elderly adults with low muscular strength. The findings support interventions to promote resistance training in older adults with low muscular strength and continuing interventional research in this space.

One limitation of this study is that the primary outcome of reduction in SBP and DBP was measured only during initial and 12-week research visits using an automated blood pressure cuff. Ambulatory or home blood pressure monitoring may provide valuable insights into blood pressure trajectories over time for future studies. Additionally, there are emerging consumer wearable devices which may be able to provide longer-term at-home blood pressure estimation in future studies, although accuracy of these technologies requires more validation before routine use [4].

An ongoing challenge in the promotion of physical activity is long-term adherence to exercise programs. In the current study, participants presented 2 times per week for PRT sessions lasting ∼45 min for 12 weeks. While there were short term benefits seen in blood pressure and muscular strength in the PRT group, it remains unclear if these gains can be sustained over the longer term. In a systematic review of 9 studies assessing whether community-based exercise interventions led to sustained effects longer than 12 months, investigators identified few studies that assessed long-term physical activity levels after an exercise intervention [5]. Excitingly, for the studies that did, physical activity levels may be sustained for up to 4 years of follow-up [5]. With the growth of artificial intelligence and consumer wearable health and fitness technology, there is also the opportunity to personalize training to improve population health. Future studies may assess whether utilizing personalized health and fitness technology after completion of a supervised exercise intervention may improve longitudinal adherence and outcomes.

A comprehensive assessment for the effect of resistance training on frailty was out of the scope of the current study but represents an important future direction. In a systematic review and meta-analysis of frailty in 62 countries across the world, the prevalence of frailty ranged from 11% in individuals 50–59+ years to 51% in individuals ≥90 years old (Fig. 1) [6]. While multiple previous studies have estimated whether different exercise protocols can benefit individuals with or at risk for frailty, the quality of evidence in these previous studies demonstrates that the benefits of exercise are often based on “low” or “very low” certainty evidence based on the GRADE (Grading of Recommendations Assessment, Development, and Evaluations) framework [7]. Studies that rigorously assess the effects of an exercise intervention protocol on frailty are notoriously difficult to design as frailty is difficult to quantify and exercise intervention trials are difficult to power and implement. The two most widely validated instruments are the deficit-accumulation frailty index and the Fried frailty phenotype [7]. In the current study, investigators required low handgrip strength in the inclusion criteria and provided repeat measures of muscular strength and the SPPB (which includes gait speed). Both handgrip strength and gait speed are included in the Fried frailty phenotype, but a formal evaluation of frailty was not performed. Given the massive public health implications of preventing frailty in the aging population, collaboration is needed among researchers with expertise in frailty and the creation of exercise intervention trials to design high-quality studies to combat this public health crisis.

The current study by Laddu et al. represents a step forward in creating exercise intervention trials in older adults (≥60 years old) with low muscular strength. Importantly, this study demonstrates feasibility of creating larger exercise intervention trials to inform public health initiatives aimed at treating and preventing frailty to promote healthy aging. Lingering areas for future research include how to maintain physical activity levels after an exercise intervention protocol, the effect of exercise intervention protocols on longer term measures of blood pressure in similar at-risk populations, and the optimal exercise protocols for the prevention and treatment of clinical frailty and optimization of cardiovascular risk. The results from this study are a useful starting point, demonstrating that for resistance training in older adults with low muscular strength, the gains are worth the strain!

## Sources of funding

None

## Disclosures

Dr. Petek’s Sports Cardiology Program receives compensation for pre-participation cardiovascular screening for the National Hockey League Scouting Combine, University of Portland, and Portland State University. Additionally, his Sports Cardiology Program has received funding from the Joel Cornette Foundation for the Outcomes Registry for Cardiac Conditions in Athletes study. Dr. Moulson reports serving on advisory boards and/or receiving honoraria from Amgen, GSK, HLS Therapeutics, Novartis, NovoNordisk, and Sanofi.

The other authors report no relevant disclosures.

## CRediT authorship contribution statement

**Bradley J. Petek:** Writing – review & editing, Writing – original draft, Conceptualization. **Nathaniel Moulson:** Writing – review & editing, Writing – original draft. **Jason V. Tso:** Writing – review & editing, Writing – original draft.

## Declaration of competing interest

The authors declare the following financial interests/personal relationships which may be considered as potential competing interests:

Nathaniel Moulson reports a relationship with Amgen Inc that includes: consulting or advisory. Nathaniel Moulson reports a relationship with GlaxoSmithKline Inc that includes: consulting or advisory. Nathaniel Moulson reports a relationship with HLS Therapeutics Inc that includes: consulting or advisory. Nathaniel Moulson reports a relationship with Novartis Pharmaceuticals that includes: consulting or advisory. Nathaniel Moulson reports a relationship with Novo Nordisk Inc that includes: consulting or advisory. Nathaniel Moulson reports a relationship with Sanofi SA that includes: consulting or advisory. Dr. Petek’s Sports Cardiology Program receives compensation for pre-participation cardiovascular screening for the National Hockey League Scouting Combine, University of Portland, and Portland State University. If there are other authors, they declare that they have no known competing financial interests or personal relationships that could have appeared to influence the work reported in this paper.
